# Supplementary material for: Effect of Bt-Cry1Ab Maize Commercialization on Arthropod Community Biodiversity in Southwest China
Source: Insects. 2025 Nov 5;16(11):1132. doi: 10.3390/insects16111132 (PMC12653073; doi:10.3390/insects16111132)
Supplement: Supplementary file 1 [file insects-16-01132-s001.zip › insects-3950711-supplementary.pdf]

Table S1. Species information, abundance and percentage of arthropod community collected in Bt and non-Bt maize fields in 2023 and 2024

Orders (17)	Families (45)	Species/types (80)	2023						2024					
			Bt maize			Non–Bt maize			Bt maize			Non–Bt maize		
			No.	Ratio (%)	Type	No.	Ratio (%)	Type	No.	Ratio (%)	Type	No.	Ratio (%)	Type
Target herbivorous (1 orders, 3 families, 15 species/types)			2457	0.87		2543	1.81		709	0.94		418	0.54	
Lepidoptera	Pyraloidea	*Ostrinia furnacalis* Guenée	31	0.01	+	718	0.51	+	679	0.90	+	305	0.40	+
		Dichocrocis punctiferalis Guenée	12	0.00	+	449	0.32	+	1	0.00	+	26	0.03	+
		Pyraloidea	17	0.01	+	57	0.04	+	0	0.00	–	0	0.00	–
	Noctuidae	*Peridroma saucia* Hübner	2148	0.76	+	701	0.50	+	0	0.00	–	1	0.00	+
		Mythimna separata Walker	22	0.01	+	236	0.17	+	3	0.00	+	27	0.04	+
		Mythimna loreyi Duponchel	162	0.06	+	272	0.19	+	26	0.03	+	42	0.05	+
		Helicoverpa armigera Hübner	61	0.02	+	83	0.06	+	0	0.00	–	16	0.02	+
		Agrotis ipsilon Hufnagel	1	0.00	+	2	0.00	+	0	0.00	–	0	0.00	–
		Agrotis segetum Denis & Schiffermüller	0	0.00	–	1	0.00	+	0	0.00	–	0	0.00	–
		Emmelia trabealis Scopoli	1	0.00	+	0	0.00	–	0	0.00	–	0	0.00	–
		Eutelia blandiatrix Guenée	1	0.00	+	0	0.00	–	0	0.00	–	0	0.00	–
		Sesamia inferens Walker	0	0.00	–	0	0.00	–	0	0.00	–	1	0.00	+
		Argyrogramma agnata Staudinger	0	0.00	–	20	0.01	+	0	0.00	–	0	0.00	–
		Noctuidae	1	0.00	+	3	0.00	+	0	0.00	–	0	0.00	–
	Lymantriidae	*Porthesia scintillans* Walker	0	0.00	–	1	0.00	+	0	0.00	–	0	0.00	–
Non–target herbivorous (7 orders, 19 families, 35 species/types)			266901	94.17		129846	92.51		72982	97.20		75468	97.88	
Hemiptera	Aphididae	Aphididae	247837	87.45	+++	128646	91.65	+++	53998	71.92	+++	48037	62.30	+++
	Delphacidae	*Laodelphax striatellus* Fallén	39	0.01	+	6	0.00	+	88	0.12	+	53	0.07	+

		Sogatella furcifera Horváth	0	0.00	–	0	0.00	–	3	0.00	+	1	0.00	+
	Cicadellidae	*Thaia rubiginosa* Kuoh	228	0.08	+	7	0.00	+	4	0.01	+	13	0.02	+
	Aleyrodidae	*Bemisia tabaci* Gennadius	27	0.01	+	2	0.00	+	0	0.00	–	0	0.00	–
	Cercopidae	*Cosmoscarta dorsimacula* Walker	0	0.00	–	0	0.00	–	0	0.00	–	1	0.00	+
	Pentatomidae	*Nezara viridula* L.	11	0.00	+	15	0.01	+	221	0.29	+	78	0.10	+
		Dolycoris baccarum L.	6	0.00	+	13	0.01	+	1	0.00	+	12	0.02	+
		Halyomorpha halys Stål	0	0.00	–	0	0.00	–	0	0.00	–	2	0.00	+
		Stollia guttiger Thunberg	10	0.00	+	5	0.00	+	0	0.00	–	0	0.00	–
		Pentatomidae	240	0.08	+	66	0.05	+	76	0.10	+	39	0.05	+
	Miridae	Apolygus lucorum Meyer–Dür.	1238	0.44	+	138	0.10	+	1	0.00	+	0	0.00	–
		Adelphocoris suturalis Jakovlev	67	0.02	+	27	0.02	+	0	0.00	–	0	0.00	–
		Adelphocoris fasciaticollis Reuter	0	0.00	–	1	0.00	+	0	0.00	–	0	0.00	–
		Trigonotylus caelestialium Kirkaldy	1	0.00	+	1	0.00	+	1	0.00	+	0	0.00	–
		Miridae	0	0.00	–	0	0.00	–	2	0.00	+	0	0.00	–
	Coreidae	*Riptortus pedestris* Fabricius	34	0.01	+	0	0.00	–	0	0.00	–	0	0.00	–
		Cletus punctiger Dallas	0	0.00	–	0	0.00	–	0	0.00	–	1	0.00	+
		Leptocorisa chinensis Dallas	0	0.00	–	0	0.00	–	11	0.01	+	1	0.00	+
		Coreidae	2	0.00	+	0	0.00	–	0	0.00	–	0	0.00	–
	Fulgoridae	*Raivuna nakanonis* Matsumura	0	0.00	–	2	0.00	+	0	0.00	–	0	0.00	–
Coleoptera	Chrysomelidae	*Monolepta signata* Olivier	42	0.01	+	13	0.01	+	0	0.00	–	0	0.00	–
		Phyllotreta striolata Fabricius	229	0.08	+	71	0.05	+	2	0.00	+	0	0.00	–
		Chrysomelidae	204	0.07	+	33	0.02	+	0	0.00	–	0	0.00	–
		Altica goeffroy	868	0.31	+	284	0.20	+	1	0.00	+	0	0.00	–
	Elateridae	Elateridae	0	0.00	–	2	0.00	+	0	0.00	–	0	0.00	–
	Curculionidae	Curculionidae	6	0.00	+	14	0.01	+	0	0.00	–	0	0.00	–

	Scarabaeidae	*Protaetia brevitarsis* Lewis	6	0.00	+	2	0.00	+	1	0.00	+	0	0.00	–
		Anomala corpulenta Motschulsky	3	0.00	+	3	0.00	+	0	0.00	–	0	0.00	–
	Cerambycidae	*Chlorophorus* sp.	1	0.00	+	2	0.00	+	0	0.00	–	0	0.00	–
Thysanoptera	Thripoidea	*Frankliniella tenuicornis* Uzel	1544	0.54	+	314	0.22	+	4	0.01	+	8	0.01	+
Orthoptera	Gryllidae	*Teleogryllus* sp.	123	0.04	+	7	0.00	+	0	0.00	–	0	0.00	–
	Oedipodidae	*Aiolopus tamulus* Fabricius	139	0.05	+	39	0.03	+	7	0.01	+	12	0.02	+
Blattaria	Blattidae	*Blattella germanica* L.	10	0.00	+	22	0.02	+	0	0.00	–	0	0.00	–
Arachnoidea	Tetranychidae	*Tetranychus cinnabarinus* Biosduval	13986	4.93	++	111	0.08	+	18561	24.72	+++	27210	35.29	+++
Nature enemy (10 orders, 17 families, 24 species/types)			8617	3.04		2323	1.65		1309	1.74		676	0.88	
Hemiptera	Reduviidae	*Sphedanolestes impressicollis* Stål	1	0.00	+	2	0.00	+	0	0.00	–	0	0.00	–
	Anthocoridae	*Orius sauteri* Poppius	1070	0.38	+	282	0.20	+	65	0.09	+	41	0.05	+
Coleoptera	Coccinellidae	*Harmonia axyridis* Palla	787	0.28	+	366	0.26	+	502	0.67	+	182	0.24	+
		Propylaea japonica Thunberg	123	0.04	+	87	0.06	+	76	0.10	+	35	0.05	+
		Menochilus sexmaculata Fabricius	3	0.00	+	0	0.00	–	1	0.00	+	0	0.00	–
		Adonia variegata Goeze	3	0.00	+	3	0.00	+	0	0.00	–	0	0.00	–
		Coccinella septempunctata L.	35	0.01	+	2	0.00	+	5	0.01	+	1	0.00	+
		Scymnus sp.	146	0.05	+	25	0.02	+	0	0.00	–	0	0.00	–
	Staphylinidae	*Paederus fuscipes* Curtis	23	0.01	+	10	0.01	+	24	0.03	+	1	0.00	+
	Cicindelidae	*Cicindela aurulenta* Fabricius	0	0.00	–	1	0.00	+	0	0.00	–	0	0.00	–
Dermaptera	Anisolabididae	*Anisolabis maritima* Borelli	39	0.01	+	36	0.03	+	17	0.02	+	10	0.01	+
Diptera	Syrphidae	Syrphidae	59	0.02	+	75	0.05	+	17	0.02	+	9	0.01	+
	Asilidae	*Cophinopoda chinensis* Fabricius	2	0.00	+	0	0.00	–	0	0.00	–	0	0.00	–
Neuroptera	Chrysopidae	*Chrysoperla sinica* Tjeder	1529	0.54	+	336	0.24	+	321	0.43	+	126	0.16	+
	Hemerobiidae	*Hemerobius humuli* L.	9	0.00	+	2	0.00	+	0	0.00	–	0	0.00	–
Hymenoptera	Ichneumonidae	*Campoletis chlorideae* Uchida	10	0.00	+	8	0.01	+	0	0.00	–	0	0.00	–

	Braconidae	*Macrocentrus cingulum* Brischke	19	0.01	+	205	0.15	+	0	0.00	–	0	0.00	–
		Lysaphidus sp.	3192	1.13	++	123	0.09	+	68	0.09	+	11	0.01	+
		Braconidae	0	0.00	–	0	0.00	–	42	0.06	+	125	0.16	+
	Scelionidae	*Telenomus remus* Nixon	244	0.09	+	91	0.06	+	0	0.00	–	0	0.00	–
Orthoptera	Tettigoniidae	*Hexacentrus* sp.	37	0.01	+	14	0.01	+	0	0.00	–	9	0.01	+
Mantodea	Mantidea	*Tenodera sinensis* Saussure	1	0.00	+	2	0.00	+	0	0.00	–	1	0.00	+
Arachnoidea	Anystidae	*Anystis* sp.	66	0.02	+	24	0.02	+	1	0.00	+	23	0.03	+
Araneae	Araneae	Araneae	1219	0.43	+	629	0.45	+	170	0.23	+	102	0.13	+
Neutral insect (5 orders, 6 families, 6 species/types)			5439	1.92		5653	4.03		85	0.11		544	0.71	
Lepidoptera	Plutellidae	*Plutella xylostella* L.	0	0.00	–	1	0.00	+	0	0.00	–	0	0.00	–
	Pieridae	*Pieris rapae* L.	62	0.02	+	0	0.00	–	0	0.00	–	6	0.01	+
Hymenoptera	Formicidae	Formicidae	5363	1.89	++	5650	4.03	++	84	0.11	+	537	0.70	+
Ephemeroptera	Heptagenioidea	*Heptagenia chinensis* Ulmer	7	0.00	+	2	0.00	+	0	0.00	–	0	0.00	–
Scutigeromorpha	Theteuonema	*Theteuonema tuberculata* Wood	0	0.00	–	0	0.00	–	1	0.00	+	0	0.00	–
Helicida	Limacidae	*Agriolimax agrestis* L.	7	0.00	+	0	0.00	–	0	0.00	–	1	0.00	+
Total (aboundance and number of species/types)			283414 (63)			140365 (63)			75085 (36)			77106 (38)		

No.: the number of individuals; +: rare species; ++: common species; +++: dominant species; –: unobserved

Table S2. Results of linear mixed-effects models comparing the abundance (individual numbers) for different arthropod groups across year, maize growth stage, and maize type (non-Bt vs. Bt)

Number of individuals	Source	Type III sum of squares	*df*	Mean squares	*F*-value	*P*-value
Arthropod community	Year	1.11	1	1.11	4.94	0.027
	Growth stage	124.03	6	20.67	91.90	< 0.001
	Maize type	0.54	1	0.54	2.38	0.124

	Year × growth stage	18.18	6	3.03	13.47	< 0.001
	Year × maize type	1.00	1	1.00	4.43	0.036
	Growth stage × maize type	2.19	6	0.36	1.62	0.142
	Year × growth stage × maize type	0.98	6	0.16	0.72	0.632
	Error	59.83	266	0.23		
	Total	2011.59	294			
Target herbivores	Year	0.65	1	0.65	2.55	0.111
	Growth stage	33.14	6	5.52	21.71	< 0.001
	Maize type	4.05	1	4.05	15.91	< 0.001
	Year × growth stage	8.46	6	1.41	5.54	< 0.001
	Year × maize type	2.93	1	2.93	11.50	0.001
	Growth stage × maize type	4.59	6	0.77	3.01	0.007
	Year × growth stage × maize type	4.01	6	0.67	2.63	0.017
	Error	67.69	266	0.25		
	Total	265.22	294			
Non-target herbivores	Year	2.19	1	2.19	6.24	0.013
	Growth stage	155.06	6	25.84	73.57	< 0.001
	Maize type	2.97	1	2.97	8.46	0.004
	Year × growth stage	23.99	6	4.00	11.38	< 0.001
	Year × maize type	2.26	1	2.26	6.43	0.012
	Growth stage × maize type	1.54	6	0.26	0.73	0.625
	Year × growth stage × maize type	1.29	6	0.22	0.61	0.722
	Error	93.45	266	0.35		
	Total	1831.14	294			
Natural enemies	Year	0.03	1	0.03	0.26	0.610
	Growth stage	31.11	6	5.19	49.72	< 0.001

	Maize type	1.13	1	1.13	10.83	0.001
	Year × growth stage	5.09	6	0.85	8.14	< 0.001
	Year × maize type	0.05	1	0.05	0.51	0.475
	Growth stage × maize type	1.69	6	0.28	2.70	0.015
	Year × growth stage × maize type	0.65	6	0.11	1.04	0.398
	Error	27.74	266	0.10		
	Total	608.34	294			
Neutral insects	Year	4.20	1	4.20	17.76	< 0.001
	Growth stage	40.93	6	6.82	28.84	< 0.001
	Maize type	1.01	1	1.01	4.26	0.040
	Year × growth stage	4.57	6	0.76	3.22	0.005
	Year × maize type	1.62	1	1.62	6.86	0.009
	Growth stage × maize type	1.96	6	0.33	1.38	0.221
	Year × growth stage × maize type	0.48	6	0.08	0.34	0.918
	Error	62.93	266	0.24		
	Total	408.24	294			

Table S3. Results of linear mixed-effects models comparing the number of species for different arthropod groups across year, maize growth stage, and maize type (non-Bt vs. Bt)

Number of species	Source	Type III sum of squares	*df*	Mean squares	*F*-value	*P*-value
Arthropod community	Year	0.25	1	0.25	21.78	< 0.001
	Growth stage	6.31	6	1.05	91.42	< 0.001
	Maize type	0.00	1	0.00	0.39	0.535

	Year × growth stage	0.87	6	0.14	12.54	< 0.001
	Year × maize type	0.01	1	0.01	0.82	0.366
	Growth stage × maize type	0.06	6	0.01	0.81	0.563
	Year × growth stage × maize type	0.02	6	0.00	0.32	0.928
	Error	3.06	266	0.01		
	Total	335.48	294			
Target herbivores	Year	0.04	1	0.04	1.34	0.249
	Growth stage	5.26	6	0.88	32.26	< 0.001
	Maize type	1.51	1	1.51	55.43	< 0.001
	Year × growth stage	0.43	6	0.07	2.65	0.016
	Year × maize type	0.06	1	0.06	2.33	0.128
	Growth stage × maize type	0.22	6	0.04	1.38	0.224
	Year × growth stage × maize type	0.10	6	0.02	0.64	0.698
	Error	7.22	266	0.03		
	Total	44.42	294			
Non-target herbivores	Year	0.18	1	0.18	10.62	0.001
	Growth stage	3.13	6	0.52	31.09	< 0.001
	Maize type	0.19	1	0.19	11.52	0.001
	Year × growth stage	0.49	6	0.08	4.84	< 0.001
	Year × maize type	0.06	1	0.06	3.49	0.063
	Growth stage × maize type	0.19	6	0.03	1.90	0.082
	Year × growth stage × maize type	0.33	6	0.05	3.25	0.004
	Error	4.46	266	0.02		
	Total	155.23	294			
Natural enemies	Year	0.06	1	0.06	2.99	0.085
	Growth stage	4.90	6	0.82	39.49	< 0.001

	Maize type	0.14	1	0.14	6.51	0.011
	Year × growth stage	1.00	6	0.17	8.03	< 0.001
	Year × maize type	0.01	1	0.01	0.30	0.582
	Growth stage × maize type	0.09	6	0.02	0.71	0.646
	Year × growth stage × maize type	0.07	6	0.01	0.59	0.736
	Error	5.51	266	0.02		
	Total	162.84	294			
Neutral insects	Year	0.09	1	0.09	8.41	0.004
	Growth stage	1.13	6	0.19	18.37	< 0.001
	Maize type	0.02	1	0.02	1.50	0.221
	Year × growth stage	0.07	6	0.01	1.20	0.305
	Year × maize type	0.15	1	0.15	14.67	< 0.001
	Growth stage × maize type	0.06	6	0.01	0.92	0.484
	Year × growth stage × maize type	0.24	6	0.04	3.89	0.001
	Error	2.72	266	0.01		
	Total	21.81	294			

Table S4. Results of linear mixed-effects models comparing the diversity indices for arthropods across year, maize growth stage, and maize type (non-Bt vs. Bt)

Diversity index	Source	Type III sum of squares	*df*	Mean squares	*F*-value	*P*-value
Simpson diversity index	Year	0.00	1	0.00	0.73	0.393
	Growth stage	0.30	6	0.05	12.43	< 0.001
	Maize type	0.00	1	0.00	0.06	0.808
	Year × growth stage	0.04	6	0.01	1.60	0.146

	Year × maize type	0.05	1	0.05	13.11	< 0.001
	Growth stage × maize type	0.02	6	0.00	0.70	0.647
	Year × growth stage × maize type	0.03	6	0.01	1.22	0.296
	Error	1.06	266	0.00		
	Total	9.54	294			
Shannon-Wiener diversity index	Year	0.13	1	0.13	9.34	0.002
	Growth stage	0.75	6	0.12	8.83	< 0.001
	Maize type	0.00	1	0.00	0.01	0.942
	Year × growth stage	0.16	6	0.03	1.93	0.076
	Year × maize type	0.15	1	0.15	10.79	0.001
	Growth stage × maize type	0.07	6	0.01	0.77	0.595
	Year × growth stage × maize type	0.15	6	0.03	1.82	0.095
	Error	3.75	266	0.01		
	Total	46.01	294			
Pielou evenness index	Year	0.00	1	0.00	0.80	0.372
	Growth stage	0.56	6	0.09	28.80	< 0.001
	Maize type	0.00	1	0.00	0.21	0.644
	Year × growth stage	0.07	6	0.01	3.43	0.003
	Year × maize type	0.04	1	0.04	11.06	0.001
	Growth stage × maize type	0.02	6	0.00	1.07	0.380
	Year × growth stage × maize type	0.02	6	0.00	1.11	0.356
	Error	0.87	266	0.00		
	Total	9.88	294			
McIntosh diversity index	Year	0.00	1	0.00	0.43	0.511

	Growth stage	0.32	6	0.05	15.68	< 0.001
	Maize type	7.08×10^{-5}	1	7.08×10^{-5}	0.02	0.885
	Year × growth stage	0.04	6	0.01	2.00	0.066
	Year × maize type	0.04	1	0.04	11.13	0.001
	Growth stage × maize type	0.02	6	0.00	1.18	0.319
	Year × growth stage × maize type	0.02	6	0.00	0.76	0.606
	Error	0.90	266	0.00		
	Total	6.05	294			

Table S5. Results of linear mixed-effects models comparing the stability indices for arthropods across year, maize growth stage, and maize type (non-Bt vs. Bt)

Stability index	Source	Type III sum of squares	*df*	Mean squares	*F*-value	*P*-value
N_d/N_p	Year	0.03	1	0.03	4.53	0.034
	Growth stage	0.01	6	0.00	0.32	0.925
	Maize type	0.02	1	0.02	3.57	0.060
	Year × growth stage	0.06	6	0.01	1.90	0.080
	Year × maize type	0.00	1	0.00	0.07	0.786
	Growth stage × maize type	0.04	6	0.01	1.08	0.375
	Year × growth stage × maize type	0.05	6	0.01	1.59	0.151
	Error	1.46	266	0.01		
	Total	2.07	294			
N_n/N_p	Year	0.02	1	0.02	1.46	0.228
	Growth stage	1.40	6	0.23	17.81	< 0.001
	Maize type	0.00	1	0.00	0.09	0.766
	Year × growth stage	0.43	6	0.07	5.48	< 0.001

	Year × maize type	0.09	1	0.09	6.59	0.011
	Growth stage × maize type	0.54	6	0.09	6.89	< 0.001
	Year × growth stage × maize type	0.42	6	0.07	5.29	< 0.001
	Error	3.48	266	0.01		
	Total	9.80	294			
S_d/S_p	Year	0.00	1	0.00	0.90	0.344
	Growth stage	0.02	6	0.00	1.27	0.273
	Maize type	0.01	1	0.01	3.17	0.076
	Year × growth stage	0.01	6	0.00	0.27	0.953
	Year × maize type	0.03	1	0.03	9.24	0.003
	Growth stage × maize type	0.03	6	0.01	1.53	0.170
	Year × growth stage × maize type	0.05	6	0.01	2.44	0.026
	Error	0.83	266	0.00		
	Total	2.15	294			
S_n/S_p	Year	0.01	1	0.01	0.61	0.437
	Growth stage	0.12	6	0.02	1.82	0.095
	Maize type	0.10	1	0.10	9.12	0.003
	Year × growth stage	0.30	6	0.05	4.34	< 0.001
	Year × maize type	0.02	1	0.02	2.14	0.145
	Growth stage × maize type	0.07	6	0.01	1.01	0.419
	Year × growth stage × maize type	0.20	6	0.03	2.98	0.008
	Error	3.01	266	0.01		
	Total	27.24	294			

Table S6. Summary of the redundancy analysis (RDA) of abundance, diversity and stability index of arthropods

Target variable	Explantory variable	R^2	Adjusted R^2	Contribution (%)	Explains (%)	Pseudo-*F*	*P*-value
Diversity index	Correlation with RDA structure						
	Growth stage	0.260	0.254	92.70	26.10	103.00	0.002
	Maize type	0.013	0.012	4.50	1.30	5.00	0.006
	Year	0.008	0.008	2.80	0.80	3.20	0.062
	Permutation test for axes						
	Axis 1	0.269	0.074	95.87	26.95	107.00	0.002
	Axis 2	0.007	0.002	2.59	0.73	2.90	0.136
	Axis 3	0.004	0.001	1.54	0.43	1.70	0.148
	All axes	0.281	0.274		79.17	37.80	0.002
Stability index	Correlation with RDA structure						
	Growth stage	0.047	0.040	73.20	4.70	14.40	0.002
	Maize type	0.007	0.006	10.50	0.70	2.10	0.094
	Year	0.010	0.009	16.30	1.00	3.20	0.014
	Permutation test for axes						
	Axis 1	0.054	0.003	84.78	5.44	16.70	0.002
	Axis 2	0.007	0.000	10.44	0.67	2.10	0.270
	Axis 3	0.003	0.000	4.78	0.31	1.00	0.448
	All axes	0.064	0.054		37.25	6.60	0.002
Abundance	Correlation with RDA structure						
	Growth stage	0.067	0.062	62.80	6.70	21.10	0.002
	Maize type	0.016	0.015	15.00	1.60	5.20	0.002
	Year	0.024	0.022	22.20	2.30	7.60	0.002
	Permutation test for axes						
	Axis 1	0.067	0.007	62.96	6.75	21.00	0.002

Axis 2	0.024	0.002	22.80	2.44	7.80	0.002
Axis 3	0.015	0.001	14.24	1.53	5.00	0.002
All axes	0.107	0.098		15.30	11.60	0.002

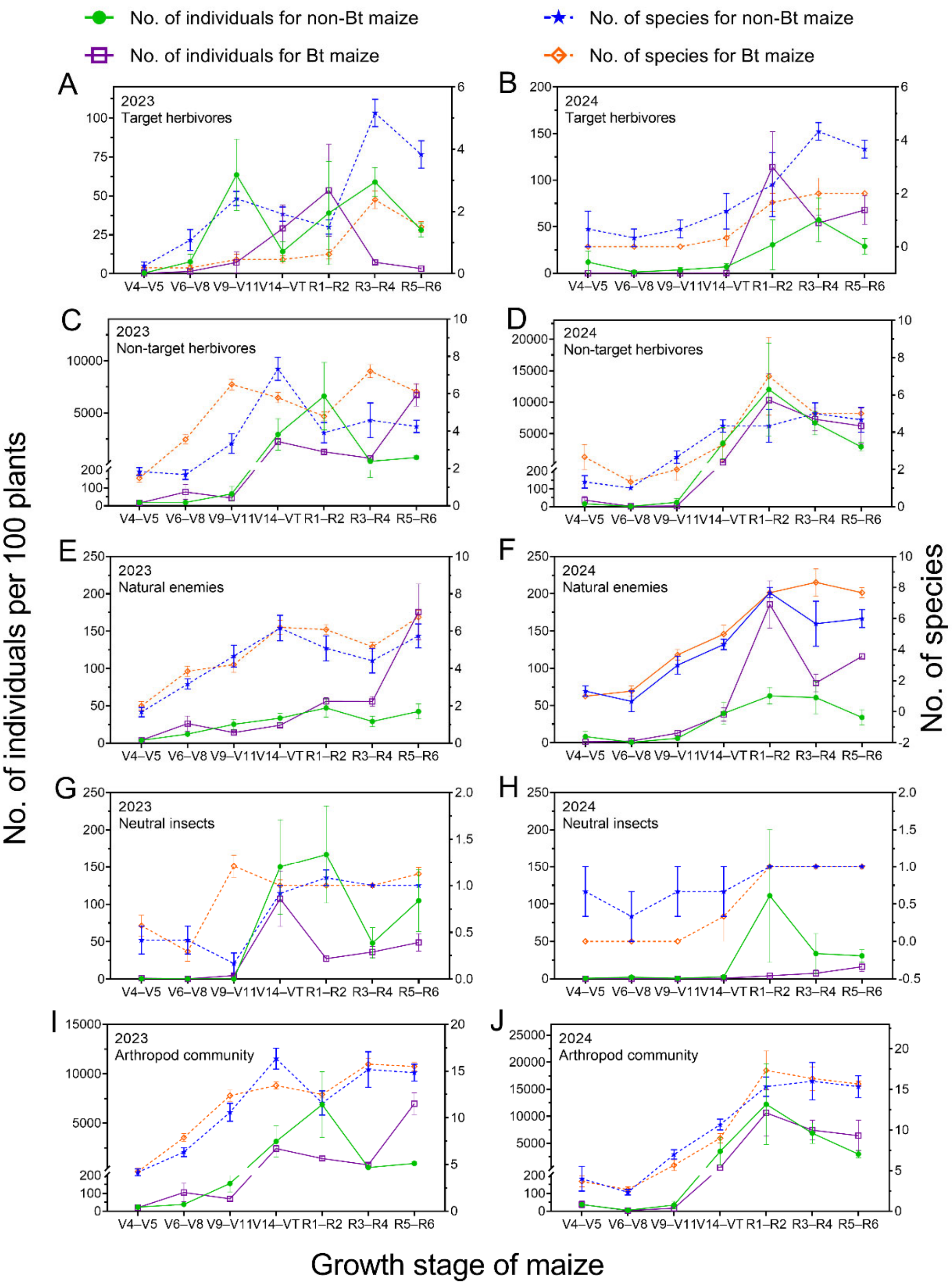

Figure S1. Number of individuals (per 100 plants) and species of four groups of arthropods in maize fields across 2023 and 2024. Data are presented as the mean ± standard error (SE). The No. represents Number.

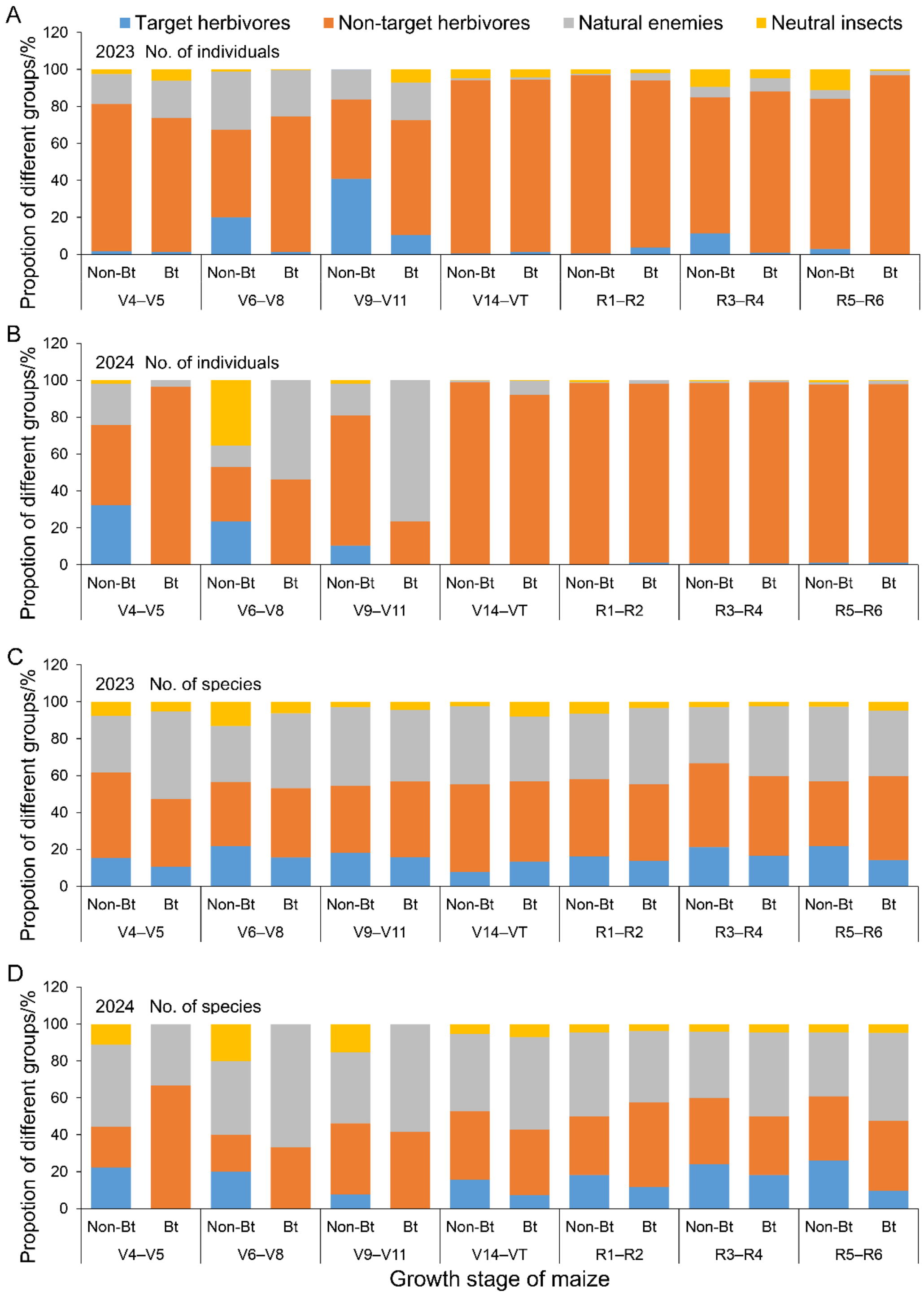

Figure S2. Proportional representation of the species number and individual number of different groups of arthropods found in Bt and non-Bt maize fields across 2023 and 2024. The No. represents Number.

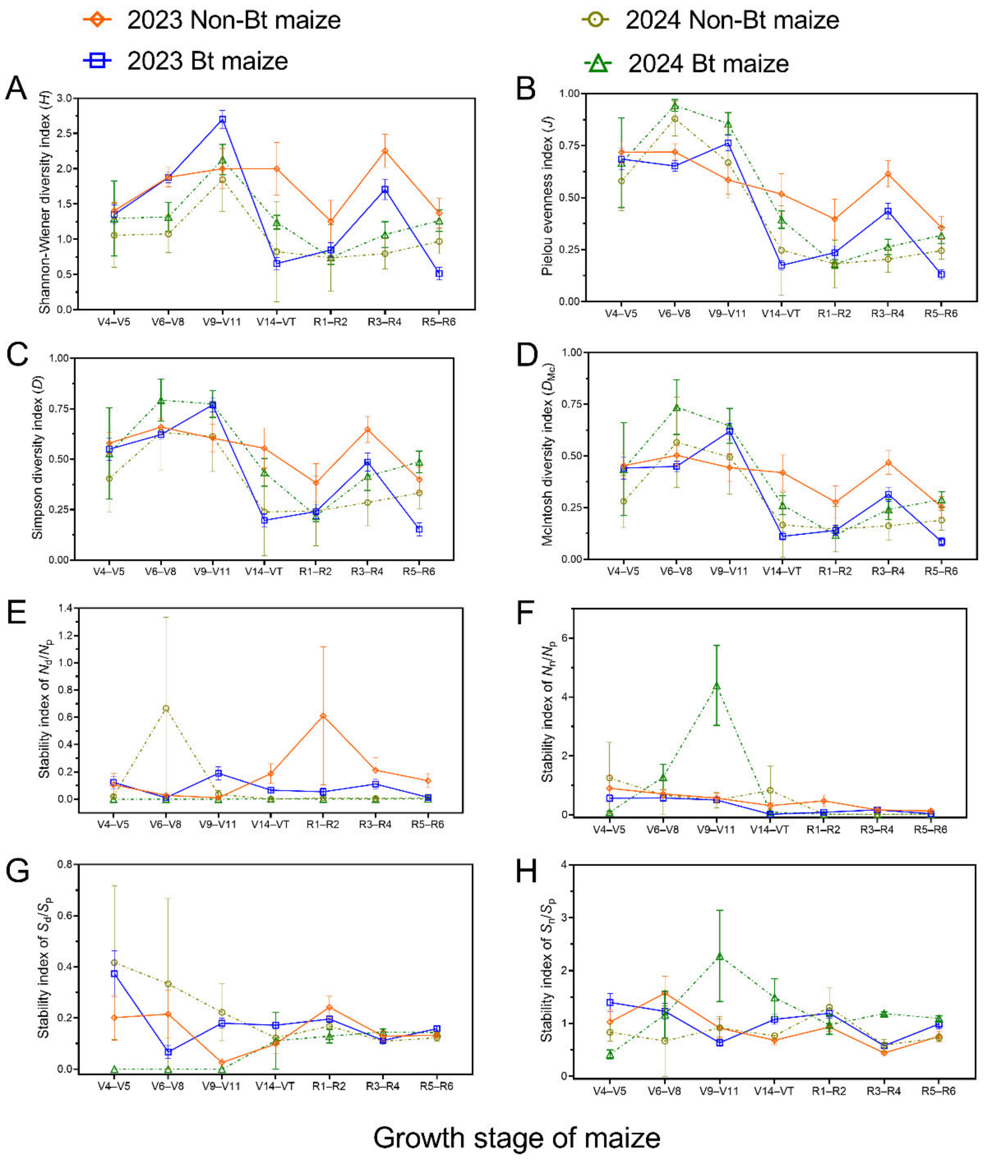

Figure S3. Diversity (A–D) and stability (E–H) indices of the arthropod community in Bt and non-Bt maize fields across 2023 and 2024. Data are presented as the mean ± SE.
